# Autonomy and refusal of dialysis: ethical and legal reflections based on the position statement of the Brazilian Society of Nephrology

**DOI:** 10.1590/2175-8239-JBN-2025-0326en

**Published:** 2026-02-06

**Authors:** Cássia Gomes da Silveira Santos, Úrsula Bueno do Prado Guirro, Luciana Dadalto

**Affiliations:** 1 Complexo Hospital de Clínicas do Paraná, Curitiba, PR, Brazil.; 2 Universidade Federal do Paraná, Curitiba, PR, Brazil.; 3 Centro Universitário Newton Paiva, Belo Horizonte, MG, Brazil.

Dear Editor,

We congratulate the publication of the **Position Statement of the Brazilian Society of Nephrology on the refusal and discontinuation of dialysis** ^1^, which represents a milestone with clinical, ethical, and social merit.

The text values Advance Care Planning, a useful tool that recognizes the process of dying as an integral part of care. The position statement reaffirms the duty to provide symptom management and to support both the patient and the family; it acknowledges that care is not solely pharmacological or interventionist, but rather multidisciplinary, comfort-focused, and grounded in the bioethical principles of beneficence and non-maleficence. Furthermore, the inclusion of Advance Directives (ADs) documents represents a significant ethical advance^2^.

However, there are points that warrant consideration: the refusal of dialysis will not always be agreed upon by the patient, family, and healthcare team. A legally competent adult is entitled to accept or refuse medical procedures. Family members authorized by the patient may express their opinions based on the patient’s values, but not on their own^3^.

Consensus-based recommendations may obscure the patient’s right to refuse treatment. The “shared decision-making” model, when misinterpreted as requiring agreement among all parties, perpetuates a conceptual misconception. Dialogue is essential; however, consensus is not a condition for legitimacy^1^,^2^. The principle of autonomy demands recognition of the individual as a moral agent capable of making decisions about their body and their own dying process. Subordinating this decision to the agreement of third parties, even if they are family members motivated by affection, constitutes a subtle form of paternalism^3^.

From a legal standpoint, the Brazilian Civil Code (Article 15) is unequivocal in providing that “no one may be compelled to undergo life-threatening medical treatment or surgical intervention.” The provision grants patients the right to refuse treatment and imposes on physicians the duty to respect such refusal, provided that it is informed and freely given^4^.

This protection is further reinforced by the Brazilian Code of Medical Ethics (Articles 22 and 31) and by Federal Council of Medicine (CFM) Resolution No. 1,995/2012 (Article 3), which establishes that ADs prevail over the wishes of family members. These regulations set limits on family interference and protect physicians who act in accordance with the patient’s wishes^5^.

Another controversial issue is the recognition of healthcare professionals’ right to conscientious objection. A physician who disagrees with the patient’s refusal of dialysis should voluntarily withdraw from assisting him or her. The absence of this reference may cause uncertainty among professionals who face value-based conflicts when dealing with decisions regarding the limitation of life-sustaining treatments^6^.

Finally, the recommendation to formalize decisions through a consent form signed by all parties warrants reconsideration. This requirement may become mere bureaucracy and convey the mistaken idea that the decision is a family or collegial concession rather than the legitimate exercise of patient autonomy. CFM Resolution No. 1,995/2012 determines that the documentation of advance directives in medical records constitutes a valid and sufficient basis for supporting the healthcare professional. Excessive formality, in contexts of suffering, may create barriers to dialogue and compassionate care^2^.

Recognizing therapeutic limits means recognizing the dignity of dying. Refusal of dialysis should be understood as a patient’s right, and physicians should understand the exercise of this right as an expression of self-determination rather than a renunciation of care^3^,^6^.

We hope that future revisions will deepen the understanding of shared decision-making not as a quest for unanimity, but as a dialogical process that respects autonomy, upholds beneficence, and protects human dignity.

## Data Availability

No new data were generated or analyzed in this study.
