# Supplementary material for: Autonomy and refusal of dialysis: ethical and legal reflections based on the position statement of the Brazilian Society of Nephrology
Source: J Bras Nefrol. 2026 Feb 6;48(2):e20250326. doi: 10.1590/2175-8239-JBN-2025-0326en (PMC12895743; doi:10.1590/2175-8239-JBN-2025-0326en)
Supplement: Translation

# Translation: Autonomia e recusa da diálise: reflexões éticas e jurídicas a partir do posicionamento da Sociedade Brasileira de Nefrologia

**DOI:** 10.1590/2175-8239-JBN-2025-0326pt

**Authors:** Cássia Gomes da Silveira Santos, Úrsula Bueno do Prado Guirro, Luciana Dadalto

**Affiliations:** 1 Complexo Hospital de Clínicas do Paraná, Curitiba, PR, Brasil.; 2 Universidade Federal do Paraná, Curitiba, PR, Brasil.; 3 Centro Universitário Newton Paiva, Belo Horizonte, MG, Brasil.

Prezado Editor,

Parabenizamos a publicação do **Posicionamento da Sociedade Brasileira de Nefrologia sobre a recusa e a descontinuação da diálise** ^1^, que representa um marco, com mérito clínico, ético e social.

O texto valoriza o Planejamento Antecipado de Cuidados, uma ferramenta útil que reconhece o processo de morrer como parte da assistência. O posicionamento reafirma o dever de proporcionar manejo de sintomas e de apoiar o paciente e a família; reconhece que o cuidado não é somente farmacológico ou intervencionista, mas, sim, multidisciplinar, focado no conforto, com premissa dos princípios bioéticos da beneficência e da não maleficência. Ademais, a inclusão dos documentos de Diretivas Antecipadas de Vontade (DAV) representa um avanço ético significativo^2^.

Entretanto, há pontos que merecem reflexão: a recusa da diálise nem sempre será consensual entre paciente, família e equipe. A pessoa maior de idade e capaz tem o direito de aceitar ou recusar procedimentos. Familiares autorizados pelo paciente poderão opinar com base nos valores do paciente, mas não nos seus próprios^3^.

A indicação consensual pode obscurecer o direito do paciente à recusa terapêutica. O modelo de “decisão compartilhada”, quando interpretado equivocadamente como necessidade de concordância entre todos, perpetua um equívoco conceitual. É essencial estabelecer o diálogo, mas o consenso não é condição de legitimidade^1^,^2^. O princípio da autonomia impõe o reconhecimento do sujeito como agente moral capaz de tomar decisões sobre o próprio corpo e o próprio morrer. Subordinar essa decisão à concordância de terceiros, mesmo que familiares movidos por afeto, configura uma forma sutil de paternalismo^3^.

Do ponto de vista jurídico, o Código Civil (Artigo 15) é inequívoco ao dispor que “ninguém pode ser constrangido a submeter-se, com risco de vida, a tratamento médico ou intervenção cirúrgica”. A norma confere ao paciente o direito de recusar tratamento e impõe ao médico o dever de respeitar tal recusa, desde que informada e livre^4^.

Essa proteção é reforçada pelo Código de Ética Médica (Artigos 22 e 31) e pela Resolução CFM nº 1.995/2012 (Artigo 3º), que determina que as DAVs prevalecem sobre a vontade dos familiares. Trata-se de normatização que estabelece o limite da interferência familiar e protege o médico que age conforme o desejo do paciente^5^.

Outro ponto polêmico é o reconhecimento do direito do profissional à objeção de consciência. Um médico que discorda da recusa à diálise deveria se afastar voluntariamente da assistência. A ausência dessa referência pode gerar insegurança em profissionais que enfrentam conflitos de valores diante de decisões de limitação terapêutica^6^.

Por fim, a recomendação de formalizar as decisões mediante termo de consentimento assinado por todas as partes merece reconsideração. Essa exigência pode se tornar mera burocracia e transmitir a equivocada ideia de que a decisão é uma concessão familiar ou colegiada, e não o exercício legítimo da autonomia do paciente. A Resolução CFM nº 1.995/2012 determina que o registro das diretivas em prontuário é documento hábil e suficiente para respaldar o profissional. O excesso de formalidade, em contextos de sofrimento, pode gerar barreiras ao diálogo e ao cuidado compassivo^2^.

Reconhecer o limite terapêutico é reconhecer a dignidade do morrer. A recusa à diálise deve ser compreendida como direito do paciente, cabendo ao médico entender o exercício desse direito como manifestação de autodeterminação, e não como abdicação do cuidado^3^,^6^.

Esperamos que futuras revisões aprofundem a compreensão da decisão compartilhada não como busca de unanimidade, mas como um processo dialógico que respeita a autonomia, sustenta a beneficência e protege a dignidade humana.

## Data Availability

Nenhum dado novo foi gerado ou analisado neste estudo.
